# Surgical Reconstruction of Abdominal Wall Endometriosis Post-Cesarean Section: A Monocentric Experience of a Rare Pathology

**DOI:** 10.3390/jcm14155416

**Published:** 2025-08-01

**Authors:** Agostino Fernicola, Armando Calogero, Gaia Peluso, Alfonso Santangelo, Domenico Santangelo, Felice Crocetto, Gianluigi Califano, Caterina Sagnelli, Annachiara Cavaliere, Antonella Sciarra, Filippo Varlese, Antonio Alvigi, Domenica Pignatelli, Federico Maria D’Alessio, Martina Sommese, Nicola Carlomagno, Michele Santangelo

**Affiliations:** 1 Department of Advanced Biomedical Sciences, Unit of Emergency Surgery, Federico II University, 80131 Naples, Italy; armando.calogero2@unina.it (A.C.); gaia.peluso@unina.it (G.P.); filippo.varlese@unina.it (F.V.); sommesemartina@gmail.com (M.S.); misantan@unina.it (M.S.); 2 Unit of Urology, Division of Oncology, Gianfranco Soldera Prostate Cancer Lab, Vita-Salute San Raffaele University, IRCCS San Raffaele, 20132 Milan, Italy; alfonso.santangelo@hsr.it; 3 Department of Radiology, Vita-Salute San Raffaele University, IRCCS San Raffaele, 20132 Milan, Italy; domenico.santangelo@hsr.it; 4 Department of Neurosciences, Reproductive Sciences and Odontostomatology, Federico II University, 80131 Naples, Italy; felice.crocetto@unina.it (F.C.);; 5 Department of Mental and Physical Health and Preventive Medicine, University of Campania “Luigi Vanvitelli”, 81100 Caserta, Italy; caterina.sagnelli@unicampania.it; 6 Plastic Surgery Unit, Federico II University, 80131 Naples, Italy; annachiara.cavaliere@unina.it; 7 Department of Precision Medicine, University of Campania “Luigi Vanvitelli”, 81100 Caserta, Italy; antonella.sciarra@unicampania.it; 8 Department of Clinical Medicine and Surgery, Federico II University, 80131 Naples, Italy; antalvigi@gmail.com (A.A.); domenicapignatelli@outlook.it (D.P.); federicom.dalessio@gmail.com (F.M.D.)

**Keywords:** abdominal wall endometriosis (AWE), abdominal wall endometriosis and surgical approach, surgical scar endometriosis, surgical management of abdominal wall endometriosis, abdominal wall endometriosis after cesarean section, cesarean section (CS)

## Abstract

**Background:** Abdominal wall endometriosis (AWE) is a rare pathological condition that mostly occurs in the post-cesarean section. This study aimed to describe the surgical approach employed in treating 31 patients at our center over the past decade and compare the outcomes with those reported in scientific literature. **Methods:** We retrospectively evaluated the data of 31 patients with a cesarean section history who underwent surgery for AWE excision between 1 November 2012, and 31 January 2023, at the University of Naples Federico II, Italy. Subsequently, we reviewed the scientific literature for all AWE-related studies published between 1 January 1995, and 31 July 2024. **Results:** Most women presented with a palpable abdominal mass (90.3%) at the previous surgical site associated with cyclic abdominal pain (80.6%) concomitant with menstruation. All patients underwent preoperative abdominal ultrasound and magnetic resonance imaging, 71% underwent computed tomography, and 32.2% received ultrasound-guided needle biopsies. Furthermore, 90.3% and 9.7% had previous Pfannenstiel and median vertical surgical incisions, respectively. All patients underwent laparotomic excision and abdominal wall reconstruction, with prosthetic reinforcement used in 73.5% of cases. No recurrent nodules were detected in any patient at the 12-month follow-up. **Conclusions:** AWE should be suspected in women with a history of cesarean section presenting with palpable, cyclically painful abdominal mass associated with the menstrual cycle. Preoperative ultrasound and magnetic resonance imaging are essential, and surgical excision must ensure clear margins. Abdominal wall reconstruction should include prosthetic reinforcement, except when the defect is minimal (≤1.5 cm). An ultrasound follow-up at 12 months is recommended to confirm the absence of recurrence.

## 1. Introduction

Endometriosis is a chronic inflammatory disease [1,2], most commonly occurring during the reproductive years; however, it is rare in prepubertal and adolescent girls (2–3%) and post-menopausal women (3–4%) [2,3,4]. Internal endometriosis or adenomyosis is characterized by ectopic endometrial tissue growth within the myometrium, diffusely or as localized adenomyomas [3,5]. Pelvic external endometriosis is characterized by ectopic endometriotic tissue in the pelvic organs or peritoneum [3,6], whereas extrapelvic endometriosis involves organs or tissues outside the pelvic cavity [3,6]. Epidemiologically, only a few women with endometriosis are diagnosed or aware of their condition [2,5,7]. Endometriosis affects 70–80% of women with infertility [2,8]. Risk factors include nulliparity, short menstrual cycles and heavy menstruation, intrauterine device use, hereditary (CYP1A1 mutations), and environmental factors such as diethylhexyl phthalate and bisphenol [9].

Endometriosis is primarily localized in the ovaries (80% unilaterally and 50% bilaterally) in superficial (nodular or microcystic lesions near the ovary wall) and deep (endometriotic cysts or endometriomas in the ovarian parenchyma) forms [4,6,10,11,12]. Other locations are near the uterine ligaments, pelvic peritoneum, recto-vaginal septum (thickened appearance), peritoneum (scarred appearance or dark red dots), uterine tubes, sigmoid colon, rectum, appendix, and small intestine [13,14,15,16,17,18,19].

Abdominal wall endometriosis (AWE) is among the rarest forms of primary endometriosis [20,21,22], accounting for 20% of cases, while most are secondary to obstetric and gynecological surgeries, including laparotomic, laparoscopic, or robotic procedures. Notably, 80% of parietal endometriosis is secondary to cesarean section (CS) [23,24]. AWE has an incidence rate of 0.03–3.5% [24]. Surgical intervention is the treatment choice when medical therapy cannot control pain or when neighboring organs are involved, leading to intestinal stenosis or hydronephrosis and potential renal damage [25]. The clinical improvements observed after medical therapy for implants located in other areas were not observed in parietal endometriosis [26]. Therefore, surgery remains the only curative treatment for AWE, including endometriosis secondary to CS, with mass removal effectively resolving chronic pain [22,27].

Laparotomic and laparoscopic surgical approaches are described in the literature, with notable differences among the various authors. However, we found no studies that precisely tailored the surgical approach to the location of the endometriotic nodule within the various layers of the abdominal wall. Most of the surgical experiences are derived from studies that do not simultaneously describe the authors’ experience, depending on the different locations of the endometriotic nodule in the abdominal wall. To our knowledge, this is the first monocentric study describing the laparotomic surgical approach for each location of the endometriotic nodule in the abdominal wall. This study aimed to present our experience with 31 consecutive AWE surgeries performed at our center and compare the surgical approach and outcomes with those in the existing literature.

## 2. Materials and Methods

### 2.1. Study Design and Patient Selection

This retrospective study included 31 female patients (aged > 18 years) who underwent surgery performed by the same surgeon at the General Surgery Unit of Federico II University Hospital (Naples, Italy) between 1 November 2012, and 31 January 2023. All participants had a preoperative diagnosis of AWE post-CS, confirmed postoperatively and histologically. We collected patient data from their medical records and surgical reports.

The selection criteria were a history of at least one CS, symptom onset post-CS, surgical excision of the mass, and endometriosis diagnosis for each lesion. Data collected for each patient include basic patient characteristics (age, age at CS, pregnancies, parity, and body mass index [BMI]); surgical history; surgical incision type; clinical presentation; diagnostic tests; dimensions, location, and number of masses; latent period (time elapsed between CS and symptom onset); type of surgery and resection margins; and possible use of a mesh for wall reconstruction.

Preoperative investigations included abdominal ultrasound (100% of patients); abdominopelvic magnetic resonance imaging (MRI, 100% of patients); abdominopelvic computed tomography (CT, 71% of patients, conducted pre-admission) was often performed prior to referral by external clinicians as a first-line evaluation for patients presenting with nonspecific abdominal wall pain or mass. In most cases, the CT scans were available upon hospital admission and were not specifically requested by our team; ultrasound-guided biopsy was conducted for 32.2% of patients pre-admission. Patients received preoperative antibiotic prophylaxis and postoperative intravenous antibiotic therapy with third-generation cephalosporins. Discharge criteria included no postoperative complications, normal diet tolerance, no nausea or vomiting, effective pain control, flatus restoration or stool passage, and patient consent for discharge. The data of each patient were collected in Excel (version 2016; Microsoft, Redmond, WA, USA) spreadsheets. We collected the same data types for each patient by performing a univariate descriptive analysis. For the quantitative variables, we calculated the mean, deviation range, and frequency using the Excel software (version 2016; Microsoft, Redmond, WA, USA) but did not perform statistical analysis for the categorical variables because the patients were all women, and we did not collect data about religion or educational qualification. Excel’s capabilities were deemed sufficient for the aim of this analysis. As this study was descriptive and retrospective in nature, no formal power calculation was performed. The sample size was determined by including all consecutive patients with AWE surgically treated at our institution over a 10-year period, reflecting the real-world prevalence of this rare condition. Given the rarity of AWE (estimated incidence 0.03–3.5%), this cohort represents a relatively large monocentric experience.

Subsequently, we reviewed scientific literature using PubMed, Web of Science, Scopus, and Embase to identify AWE-related studies published between 1 January 1995, and 31 July 2024. The keywords included “abdominal wall endometriosis”, “endometriosis in abdominal scars”, “endometriosis of the abdominal wall”, “abdominal wall endometriosis following cesarean section”, “abdominal wall endometriosis in the cesarean section”, “surgical approach of abdominal wall endometriosis”, and “surgical scar endometriosis”. Only English studies were included, while incomplete and non-peer-reviewed preprints were excluded. We focused on randomized controlled trials, meta-analyses, systematic reviews, and observational cohort studies. The included articles were either prospective or retrospective, monocentric or multicenter studies, and varied in patient numbers (tens or hundreds). Articles considered had to include at least one diagnostic or therapeutic approach to AWE. We excluded duplicated articles or those with unavailable full text. Furthermore, we screened the scientific literature first by title and abstract, followed by analyzing the full text. All patients provided informed consent for inclusion before participation. This study was conducted following the Declaration of Helsinki. Ethics approval from the Ethics Committee was not required, as this is not an experimental study. The Level of Evidence for this study is III, as it is a retrospective cohort study.

### 2.2. Surgical Procedures

**Figure 1:**
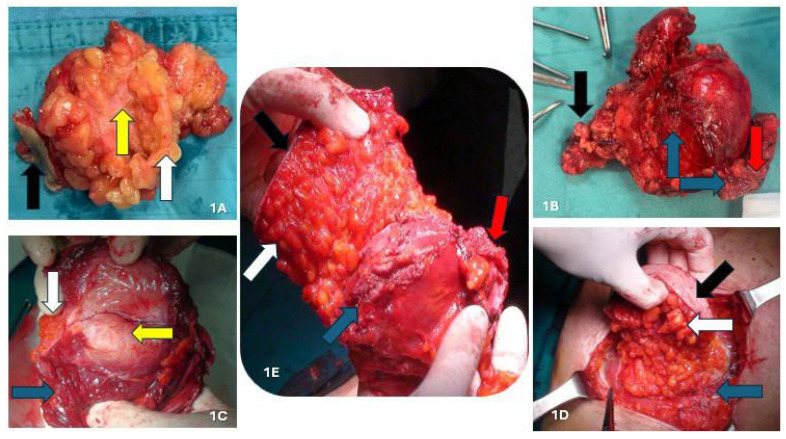
(**A**) AWE nodule located in the subcutis; (**B**) intraparietal AWE nodule involving muscular tissue; (**C**) detail of muscle involvement; (**D**) intraparietal AWE nodule involving the anterior fascia of the rectus abdominis muscles; (**E**) AWE nodule involving peritoneum. Black arrow: skin; white arrow: subcutaneous tissue; blue arrow: muscle fascia and/or muscle tissue; red arrow: peritoneum. yellow arrow: endometriotic nodule.

**Figure 2:**
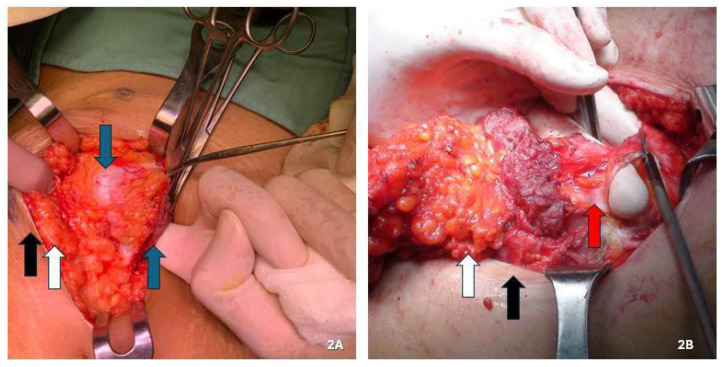
(**A**,**B**) Maneuver to open the peritoneal cavity away from the lesion to minimize the risk of cellular dissemination in order to assess the full thickness of the abdominal wall, the depth and extent of the lesion, and the extent of resection needed, so as to save as much healthy tissue as possible. (**A**) Endometriotic nodule involving only the muscular plane. (**B**) Endometriotic nodule also involving the peritoneum. Black arrow: skin; white arrow: subcutaneous tissue; blue arrow: muscle fascia and/or muscle tissue; red arrow: peritoneum.

We removed 34 endometriotic nodules from 31 patients under neuraxial anesthesia combined with sedation (Richmond Agitation Sedation Score Scale: −2) and hemodynamic monitoring. The surgical procedure was lesion depth-dependent, while the affected portion of the scar was always removed en bloc, ensuring a safety margin. Subsequently, the resection was expanded to encompass the full extent of the lesion, and surgical excision was performed radically, completely removing the lesions. Regarding the three nodules located solely in the subcutaneous plane (Figure 1A), without musculoaponeurotic layer involvement, we performed a diamond-shaped incision, including skin and subcutis, while sparing the fascial plane. No prosthetic mesh was used in these cases. We performed the same procedure previously described for the 18 nodules located between the adipose fascial planes, with minimal involvement of the aponeurotic planes. However, the anterior fascia (<1–1.5 cm) was also removed to facilitate direct reconstruction. The posterior fascia and a substantial portion of the muscles were preserved. In cases requiring anterior fascia removal of ≥1.5 cm, the abdominal wall was reconstructed using a polypropylene prosthetic mesh. Regarding lesions infiltrating the muscular plane (nine nodules), we excised the affected muscle portion, preserving the posterior fascia and peritoneum (Figure 1B–D and Figure 2A). In these cases, we used a polypropylene prosthetic mesh, positioned extraperiotoneally.

**Figure 3:**
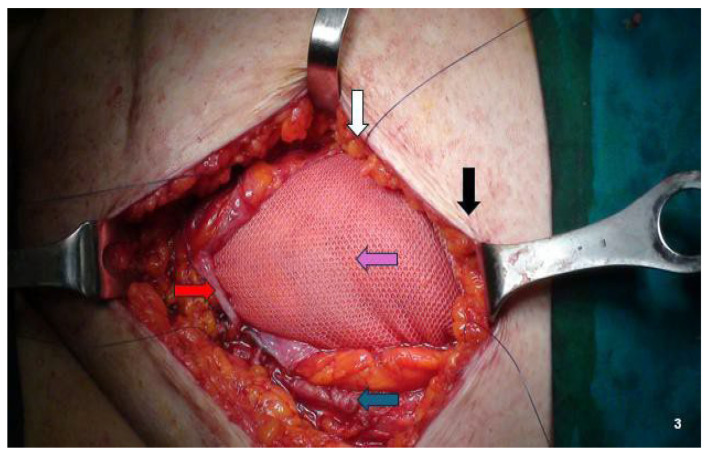
Abdominal wall reconstruction using dual mesh (non-absorbable monofilament—generally polypropylene—and absorbable monofilament—generally polycaprolactone—on external layer and a film composed of an absorbable synthetic copolymer on peritoneal layer). Black arrow: skin; white arrow: subcutaneous tissue; blue arrow: Muscle fascia and/or muscle tissue; red arrow: peritoneum. Purple arrow: prosthetic mesh.

For lesions infiltrating the peritoneum (four nodules), we performed en bloc abdominal wall amputations from the skin to the parietal peritoneum (Figure 1E and Figure 2B). We reconstructed the abdominal wall in these cases using an intraperitoneal dual mesh, which was covered with muscle, fascia, and subcutaneous tissue (Figure 3). Furthermore, we cleaned the pelvic cavity in cases of simultaneous involvement of the parietal peritoneum and pelvic endometriosis (one case each in the right ovary and uterosacral ligament). Throughout the resections, we used ultrasound or radiofrequency instruments to ensure optimal hemostasis and lymphostasis. At the end of each procedure, we always placed positive suction drains to minimize the risk of seromas and hematomas. Specifically, we used subcutaneous and periprosthetic drains when a prosthesis was used and a single subcutaneous drain when no prosthesis was employed. Prosthetic reinforcement was used when the anterior fascial defect exceeded 1.5 cm or when the lesion involved the muscular or peritoneal layers. In contrast, no mesh was used when nodules were confined to the adipose tissue or caused minimal fascial involvement (≤1.5 cm). In cases requiring intraperitoneal mesh placement, a composite dual-layer mesh was employed. These meshes consisted of a non-absorbable monofilament—typically polypropylene—and an absorbable monofilament—generally polycaprolactone—on the external surface, while the peritoneal-facing surface comprised a film of absorbable synthetic copolymer. The fixation technique was also standardized: fascial anchoring was achieved using interrupted non-absorbable sutures, whereas absorbable sutures were used for subcutaneous closure. Mesh fixation was performed under direct visualization. Mesh fixation was performed using interrupted non-absorbable sutures to the fascia under direct visualization. Subcutaneous closure was achieved with absorbable monofilament sutures.

## 3. Results

Overall, 31 females who underwent consecutive surgery for AWE at a single center and by the same surgical team were enrolled. All patients had a history of at least one CS, at least one endometriotic nodule of the abdominal wall, and a histological diagnosis of endometriosis. Preoperatively, all patients underwent abdominal ultrasound and abdominopelvic MRI. Abdominopelvic CT was performed in 22 patients (71%). Ten patients (32.2%) underwent an ultrasound-guided needle biopsy. Table 1 presents the main characteristics of the 31 patients.

Most patients (58%) were overweight. All patients had a history of CS, with 25, 5, and 1 having undergone 1, 2, and 3 CS. The latency period was the time between CS and symptom onset. Table 2 summarizes the main symptoms presented by patients.

**Figure 4:**
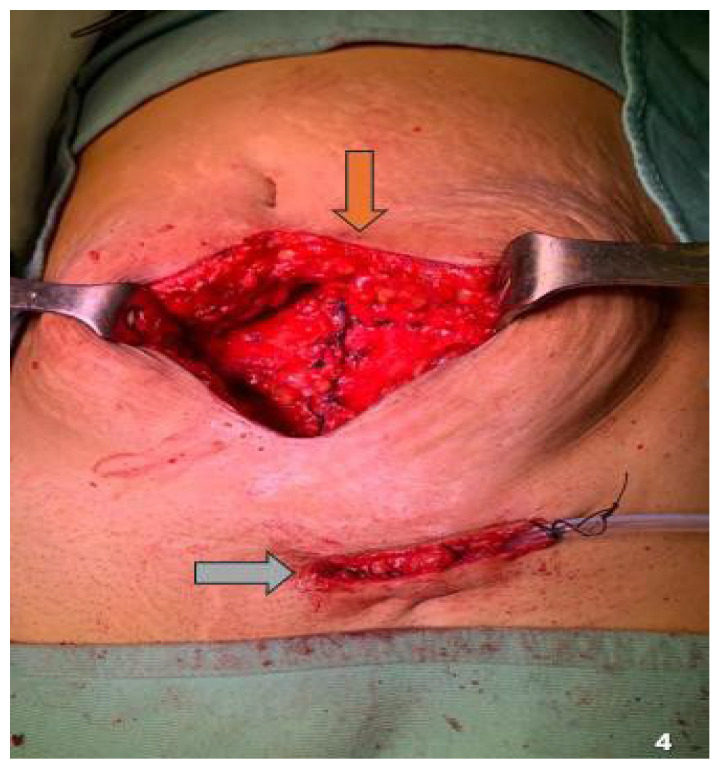
Double localization of the endometriotic nodule. Brown arrow: midline localization; gray arrow: inguinal localization.

Twenty-eight patients (90.3%) underwent CS using a Pfannenstiel incision, while three (9.7%) had a history of a median vertical incision owing to prior pelvic surgery with vertical access. Among those with Pfannenstiel incisions, the nodule was mostly located at a corner of the scar (74.2%). The number of nodules per patient ranged from 1 to 2, with 34 removed from the abdominal wall. Overall, 90.3% (n = 28) and 9.7% (n = 3) of patients had single and multiple nodules (Figure 4), respectively. All three patients with multiple nodules had previously undergone a Pfannenstiel incision. All 34 nodules were located near the incisional scar, with a mean size of 3.2 (range, 1.5–5.5) cm. Table 3 summarizes the characteristics and location of the nodules and surgical incision type.

To better describe the implants’ location in the abdominal wall, we defined a “deep limit” and “superficial limit” for each nodule. The abdominal wall was categorized by depth planes into adipose, fascial, muscular, and peritoneal planes. Among the implants, 52.9% (n = 18) were located between the adipose and fascial planes, 14.7% (n = 5) between the adipose and muscular planes, 11.8% (n = 4) between the fascial and muscular planes, 8.8% (n = 3) in the adipose layer, 5.9% (n = 2) between the fascial and peritoneal planes, and 5.9% (n = 2) between the muscular and peritoneal planes. Table 4 summarizes the patient symptoms, number of nodules per patient, polypropylene prosthesis positioning, and free margins.

Two patients (6.5%) with parietal endometriosis had concomitant pelvic endometriosis, with their parietal endometriotic nodules affecting the peritoneal plane. In all cases, the choice treatment was laparotomic surgical excision, performed by the same surgical team. Postoperatively, all patients had free resection margins. Histopathological examination revealed free resection margins ≤ 1 and ≥1 cm in 67.6% (n = 23) and 32.4% (n = 11) of nodules, respectively. A 12-month postoperative ultrasound follow-up revealed no signs of recurrence in any patient. Abdominal wall reconstruction was conducted using a mesh in 25 of the 34 nodules (73.5%): intraperitoneal dual mesh was used in four cases, and polypropylene mesh was applied in the intraparietal area for 21 nodules. Mesh was not used in nine nodules (26.5%): three cases where the nodule was confined to the adipose layer and six where the nodule infiltrated the adipose layer and partly involved (<1.5 cm) the anterior band of the rectus abdominis muscles. Furthermore, the nodules were <2 cm when no mesh was used. No incisional hernia cases were observed during the 12-month ultrasound follow-up.

## 4. Discussion

AWE is a relatively common pathological condition that primarily occurs post-CS [28,29,30,31,32]. Studies reveal that CS is responsible for 57–92% of AWE cases [3,5,23,26,32,33,34,35,36,37]. Ananias et al. found that 260 of 268 patients with AWE experienced endometriosis post-CS [35]. Here, we analyzed a series of 31 patients who underwent surgical treatment for AWE over the past decade. All cases were secondary to CS, regardless of the incision type. Specifically, 3 (9.7%) and 28 (90.3%) patients had midline vertical and Pfannenstiel incisions, respectively. No cases of AWE were observed in patients without CS history during this period, reinforcing CS as the primary risk factor. AWE predominantly affects women of childbearing age [23,38,39,40]. In the literature, the mean age of patients with AWE is between 32 and 36 years, consistent with that reported in our series (32.2 ± 6.4 years) [22,23,41,42,43,44,45]. Zhang et al. revealed that the mean latency period of AWE diagnosis is 31.6 ± 23.9 months [27], similar to the 31.7 ± 20.9 months in our series. In our experience, a high BMI correlates with an increased risk of AWE, as the greater surgical challenges encountered in corpulent patients and the high thickness of the parietal planes may facilitate cellular dissemination. Our patients had a mean BMI of 26.5 kg/m^2^. However, we found no studies in the literature correlating AWE with BMI.

AWE can present with varying symptoms and may not always be clinically evident [22,26,27,46,47,48]. In our series, almost all patients (28 cases, 90.3%) had at least one palpable and painful abdominal nodule, and 25 (80.6%) reported cyclical pain associated with menstruation, aligning with the existing literature [28,32,33,34,36]. Zhang et al. analyzed 198 endometriosis cases post-CS and identified palpable abdominal mass (98.5%) and cyclical pain (86.9%) as the most common symptoms [27]. The presence of a palpable mass in the abdominal wall requires differential diagnosis with other pathologies, including parietal abscess, lipoma, hematoma, incisional hernia, granuloma, metastases, and malignant tumors, particularly sarcomas [30,31,33,34,36].

All patients in our study underwent abdominal wall ultrasound as a first-level investigation. In 22 cases (71%), the ultrasound was followed by CT. MRI with intravenous contrast was performed in all patients as a second- and third-level examination in 9 and 22 cases, respectively. According to Foley et al., an MRI should be performed for endometriotic nodular lesions > 3 cm to guide optimal surgical planning [37]. Abdominal ultrasound mainly provides information regarding mass extension and consistency, making it the most commonly used imaging tool for first-level investigation and postoperative follow-up [26,40]. MRI provides more accurate data on the infiltration/extension in the parietal planes and the mass content and nature, making it the gold standard for evaluating soft tissue pathologies [26]. Yarmish et al. retrospectively reviewed CT findings, proposing a central soft tissue nodule with homogeneous density and linear infiltration as AWE risk factors [38].

According to most authors, including Welch et al. of the Mayo Clinic, CT is insensitive and partly useful in assessing AWE extent and staging, with MRI being superior [39]. Bartlett et al. demonstrated that MRI provides the highest AWE detection rate and the best anatomical delineation of endometriotic nodules [40]. In our opinion, CT does not provide additional information compared to MRI and is only necessary in selected cases. The high number of CT scans in our series is explained by the fact that many patients underwent CT before being referred to our center. These scans were usually requested by general practitioners or gynecologists as part of an initial diagnostic work-up for nonspecific symptoms. In our experience, however, CT does not provide any significant added value compared to MRI in the preoperative planning of AWE and is not considered necessary when high-quality MRI is available. We performed ultrasound-guided cutting needle biopsies in 32.3% of cases (n = 10), and the findings suggested endometriosis.

Although needle biopsy is a useful diagnostic tool, its role is still debated because, although it may help confirm the diagnosis, it also carries a theoretical risk of seeding endometrial cells along the needle path [35]. Moreover, a risk of pathological cell dissemination exists, particularly in cases of suspected differential diagnosis with malignancies and endometriotic nodules [48]. In our series, biopsies were performed in 64.5% of cases; however, many of these were done prior to referral, often when AWE was not yet suspected or when imaging findings were non-specific. Based on our institutional experience, we do not recommend routine biopsy in cases where the clinical history (e.g., post-cesarean scar) and imaging findings (MRI) are strongly suggestive of AWE. However, we believe biopsy may be justified in specific situations: when there is no prior gynecologic surgery and AWE is not the leading diagnosis; when MRI is inconclusive or raises suspicion for other soft-tissue tumors or in patients with atypical presentations or lesion location. In such cases, fine-needle aspiration or core biopsy may guide treatment planning, although the decision must balance diagnostic benefit against the risk of disease spread.

Considering this risk and the superficial nature of AWE lesions, directly conducting an exeretic surgery, which has diagnostic and therapeutic purposes, is a feasible approach with an adequate safety margin. From this perspective, we prefer to intervene directly on the lesion, avoiding needle biopsy. Therefore, the choice treatment is surgical excision [46,47,48]. The surgery must be exeretic and radical, ensuring complete removal of the entire lesion. Endometriotic nodule removal can be performed by laparotomy, laparoscopy, or percutaneous cryoablation [39]. Some authors prefer cryoablation in selected AWE cases to reduce the possible complications related to prosthetic mesh positioning and abdominal wall reconstruction [42,43]. Maillot et al. found a reduction in postoperative pain and shorter hospital stays [44]. However, this therapeutic approach is still under evaluation [44]. Instead, authors advocating for a laparoscopic approach to AWE treatment believe that using an Endobag (Covidien, PLC) for removing endometriotic nodules, washing the abdominal cavity, and exsufflation may prevent further endometriotic implants [45]. Denton et al. reported 16 cases in the literature [45]. Although rare, laparoscopy may be associated with endometriotic implant development at the trocar access site [45]. In such cases, the treatment is the wide excision of the site, ensuring at least a 1 cm free margin around the endometriotic lesion [45]. Therefore, we opted for a laparotomic approach in AWE treatment for these reasons and to standardize the surgical technique for endometriotic nodules located exclusively in the adipose tissue.

Doroftei et al. recommend wide excision of the lesion with at least a 1 cm safety margin to avoid recurrence [35]. However, other authors recommend a margin of 0.5–1 cm [46]. In our series, all resected nodules had clear margins, confirmed by histopathology. Although 67.6% (n = 23) of the nodules had resection margins ≤ 1 cm, we observed no recurrence at the 12-month clinical and ultrasound follow-up. This is in line with other reports in the literature, such as Doroftei et al. [35] and Zhang et al. [27], who also described recurrence-free outcomes in patients with histologically negative but narrow margins. In our series, all lesions were macroscopically well circumscribed and completely excised (R0), with no intraoperative evidence of infiltration beyond the visible capsule. We therefore believe that margins ≤ 1 cm may be acceptable in such cases, provided that radicality is confirmed both macroscopically and histologically.

Based on our experience, and in agreement with other studies, we believe that a margin of ≤1 cm is acceptable for benign and well-circumscribed lesions that respond well to hormonal treatment, provided the lesion is macroscopically free from pathological tissue and confirmed histologically. Suppressive hormonal therapy lasting 6 months may be considered if histopathology indicates positive resection margins. Thirty-four nodules were excised, with a mean size of 3.2 (range, 1.5–5.5) cm. All nodules were located near the incisional scar. Specifically, in 74.2% of nodules secondary to CS with Pfannenstiel incisions, the mass was in the incisional scar corner, consistent with previous studies, where 83% of the nodules that appeared after the Pfannenstiel incision were in the scar corner [28,30,31,33,34,35,48]. The left corner was most affected, probably because of the predominance of right-handed surgeons who positioned themselves to the left of the patient during surgery for convenience. This position may facilitate the dissemination of endometrial cells/contamination of the parietal planes from the left side during operative maneuvers, and fetal extraction from the uterine cavity.

Incisional hernia risk after excising a parietal endometriotic lesion is substantial and closely linked to the quantity of musculoaponeurotic structures removed with the nodule [34]. Therefore, correct demolitive and reconstructive surgery is essential to mitigate this risk [31]. The demolition aspect has been previously mentioned. Based on our experience and data from the literature, we recommend using a prosthesis for the reconstructive aspect to repair wall defects resulting from endometriotic nodule excision, except in cases where the nodule is confined to the adipose layer or only partly affects the anterior fascia with a defect ≤ 1.5 cm. According to Benedetto et al., patients with nodules measuring 3–5 cm underwent abdominal wall reconstruction using direct plastics without prostheses [46]. In this series, 18.2% of patients had an incisional hernia in the postoperative follow-up. Abdominal wall reconstruction using mesh was performed in our series for all 25 of the 34 (73.5%) nodules involving the musculoaponeurotic layer. Specifically, intraperitoneal dual mesh was used in the four cases with peritoneal involvement, while propylene mesh was used in the intraparietal area in the other 21 cases. In the nine cases (26.5%) where no mesh was used, nodules were confined to the adipose layer in three and minimally infiltrated the musculoaponeurotic structures in the other six (located in the subcutaneous tissue), requiring the excision of a minute part (<1.5 cm). None of these cases developed an incisional hernia at the 12-month ultrasound follow-up. In our experience, accessing the peritoneal cavity during surgery is always beneficial, except when the nodule affects only the adipose layer. In all other cases, opening the peritoneal cavity allows the surgeon to assess the abdominal wall’s entire thickness, more precisely evaluate lesion depth and extension, and determine the necessary resection extent to save as much healthy tissue as possible (Figure 2A,B). Notably, the peritoneum is opened away from the lesion to minimize cell dissemination risk.

One of the risks of exeretic interventions is blood formation or serum collections (hematoma or seromas) within the wound or near the prosthesis when used [27,47]. These collections may become infected, potentially compromising surgical outcomes [36]. To mitigate this risk, placing positive suction drains at the end of each operation (specifically near the prosthesis), applying compressive dressings, advising patients to wear elastic compression bands/briefs for extended periods, and setting adequate antibiotic therapy are good practices [27,47]. In our series, no infectious complications occurred. Drains were removed between postoperative days 3 and 5 in all patients, except for eight, in whom the drains were removed on day 7. Antibiotics were administered throughout the drainage durations. These procedures helped prevent hematomas and seromas. To further minimize intra-parietal collections, the most critical complications affecting surgical success, and to ensure effective hemostasis and lymphostasis, we used normal surgical procedures (ligatures, pierced stitches, electrosurgery, and bipolar electrocoagulation) and advanced energy devices, preferring radiofrequency-based devices to ultrasound ones because they ensure a safer and more effective vessel sectioning and coagulation, even for large- caliber vessels. Our review suggests that a laparoscopic approach is more beneficial when AWE is associated with other peritoneal endometriotic localizations or additional abdominal pathology requiring surgical treatment. Parietal reconstruction is similar to that for laparoceles performed laparoscopically. However, it is limited by the prolonged operative and general anesthesia durations.

Despite efforts to provide a rigorous methodology, this study has some limitations. First, the sample size was relatively small, and the surgical experience was based on the same surgical team at a single hospital over the past decade. Furthermore, the technique was always laparotomic; therefore, we could not compare this approach with the potential laparoscopic one. However, we selected a laparotomic approach for all 31 consecutive patients with AWE to minimize patient selection bias. In fact, laparoscopy would not have been suitable for patients with endometriotic nodules confined to the adipose tissue. Second, the follow-up period was 12 months postoperatively; therefore, a multicenter study comparing laparotomic and laparoscopic surgical approaches on patients with AWE, with extended follow-up periods, would be valuable for future research. Despite the abovementioned limitations, our surgical experience contributes to the search for the best surgical approach for a rare pathology such as AWE. Notably, we observed no recurrences or laparoceles during the 12-month follow-up, suggesting that no recurrence or incisional hernia was observed within the first 12 months, which is reassuring.

However, we recognize that 12 months may not be sufficient to exclude late recurrences of AWE, as endometriotic implants can remain quiescent for extended periods. For this reason, we are continuing to monitor this patient cohort beyond 12 months and are committed to collecting and reporting long-term follow-up data in future studies. Finally, we recognize that the absence of a control group and the lack of a comparative laparoscopic arm represent significant limitations. The exclusive use of laparotomy in this study was intended to standardize the technique and reduce selection bias. However, future prospective multicenter studies comparing laparotomic, laparoscopic, and potentially robotic approaches would be necessary to determine the most effective and least invasive strategy.

Additionally, while our cohort was limited to 31 patients, this reflects the maximum number of eligible cases over a decade at a tertiary referral center. Larger sample sizes will require collaborative registries or national databases to enable meaningful comparative analysis.

## 5. Conclusions

AWE typically develops post-CS. Women presenting with palpable and cyclically tender abdominal mass associated with the menstrual cycle should always be evaluated for AWE as part of the differential diagnosis. AWE diagnosis is based on preoperative abdominal ultrasound and MRI, as well as the patient anamnestic and symptomatic history. Surgical interventions must involve total excision of the endometriotic nodule, ensuring that margins are endometriosis-free (even < 1 cm seems to be an adequate margin). Abdominal wall reconstruction using prostheses should follow excision, except when the nodule is confined to the adipose layer or only partly affects the anterior fascia with a defect ≤ 1.5 cm. Ultrasound follow-up should be performed 12 months post-excision. For optimal outcomes, advanced energy devices should be used during nodule excision. Opening the abdominal cavity can help identify affected parietal planes, simplifying both exeresis and subsequent abdominal wall reconstruction. Moreover, future multicenter studies with larger cohorts and extended follow-up periods are necessary.

## Figures and Tables

**Table 1:** The main characteristics of the 31 patients.

Characteristics	Mean Standard ± Deviation (Range)
**Age**	32.2 ± 6.4 (18–43)
**Age at C-section**	29 ± 5.7 (17–40)
**Pregnancies**	1.8 ± 0.8 (1–4)
**Parity**	1.4 ± 0.6 (1–3)
**BMI**	25.6 ± 4.2 (18.8–34.5)
**Latency period (months)**	31.7 ± 20.9 (3–85)

**Table 2:** The main symptoms presented by patients.

Symptoms	Number (%)
**Abdominal wall mass (palpable nodule)**	28 (90.3%)
**Cyclical pain at the abdominal wall**	25 (80.6%)

**Table 3:** The location of the nodules and the type of surgical incision.

Incision Type/Position	Number of Nodules	Percentage (%)
*Pfannenstiel incision*		
Total nodules (excluding midline incision)	31	91.1% (of 34)
–Left corner	16	51.6% (of 31)
–Middle line	8	25.8% (of 31)
–Right corner	7	22.6% (of 31)
*Middle vertical incision*		
Total nodules (excluding Pfannenstiel incision)	3	8.9% (of 34)
–Upper corner	0	0.0% (of 3)
–Middle line	2	66.7% (of 3)
–Lower corner	1	33.3% (of 3)

**Table 4:** Summary of our results obtained—NR: no recurrence; PF: Pfannenstiel; IVM: middle vertical; US: ultrasound; CT: computed tomography; MR: magnetic resonance; EX: excision; PP: polypropylene mesh; DM: dual mesh.; CM: centimeters.

Case	Age	Main Symptoms	Surgical History	Number of Nodules for each Patient	Dimension of the Nodules	Imaging	Surgery	Mash	Border	Follow-up (12 Months)
**1**	30	Palpable mass. Cyclical pain	PF	1	1.8 CM	US, CT, MR	EX	NO	Free ≥1 cm	NR
**2**	22	Palpable mass. Cyclical pain	PF	1	3.6 CM	US, MR	EX	PP	Free ≤1 cm	NR
**3**	29	Palpable mass. Cyclical pain	PF	1	2.1 CM	US, MR	EX	PP	Free ≥1 cm	NR
**4**	35	Palpable mass. Cyclical pain	PF	1	1.6 CM	US, CT, MR	EX	NO	Free ≤1 cm	NR
**5**	37	Palpable mass. Cyclical pain	2 PF	1	4.5 CM	US, CT, MR	EX	DM	Free ≤1 cm	NR
**6**	24	Palpable mass. Cyclical pain	PF	1	1.5 CM	US, CT, MR	EX	PP	Free ≥1 cm	NR
**7**	18	Palpable mass. Cyclical pain	PF	1	2.2 CM	US, MR	EX	PP	Free ≥ 1 cm	NR
**8**	40	Palpable mass. Cyclical pain	IVM	1	3.3 CM	US, CT, MR	EX	PP	Free ≤1 cm	NR
**9**	37	Palpable mass. Cyclical pain	PF	1	3.1 CM	US, MR	EX	PP	Free ≤1 cm	NR
**10**	38	Palpable mass. Cyclical pain	2 PF	1	4 CM	US, CT, MR	EX	PP	Free ≤1 cm	NR
**11**	33	Palpable mass. Cyclical pain	PF	1	2.9 CM	US, CT, MR	EX	PP	free≥1 cm	NR
**12**	32	Palpable mass. Cyclical pain	2 PF	1	2.6 CM	US, CT, MR	EX	DM	Free ≤1 cm	NR
**13**	34	Palpable mass. Cyclical pain	PF	1	3.2 CM	US, CT, MR	EX	PP	Free ≥1 cm	NR
**14**	30	Palpable mass. Cyclical pain	PF	1	3.5 CM	US, CT, MR	EX	PP	Free ≤1 cm	NR
**15**	28	Palpable mass. Cyclical pain	PF	1	1.9 CM	US, MR	EX	PP	Free ≤1 cm	NR
**16**	29	Palpable mass. Cyclical pain	PF	1	3.6 CM	US, CT, MR	EX	PP	Free ≥1 cm	NR
**17**	43	Palpable mass. Cyclical pain	1 IVM	1	5.5 CM	US, CT, MR	EX	PP	Free ≤1 cm	NR
**18**	36	Palpable mass. Cyclical pain	PF	2	1.5 CM 1.8 CM	US, CT, MR	EX	NO	Free ≤1 cm	NR
**19**	34	Palpable mass. Cyclical pain	PF	1	1.5 CM	US, CT, MR	EX	NO	Free ≥1 cm	NR
**20**	35	Palpable mass. Cyclical pain	PF	1	5.2 CM	US, CT, MR	EX	PP	Free ≤1 cm	NR
**21**	39	Palpable mass. Cyclical pain	2 PF	2	2.3 CM 2.7 CM	US, CT, MR	EX	PP	Free ≤1 cm	NR
**22**	31	Palpable mass. Cyclical pain	PF	1	3.8 CM	US, CT, MR	EX	PP	Free ≥1 cm	NR
**23**	21	Palpable mass. Cyclical pain	PF	1	1.7 CM	US, MR	EX	NO	Free ≤1 cm	NR
**24**	20	Palpable mass. Cyclical pain	PF	2	1.8 CM 1.5 CM	US, CT, MR	EX	NO	Free ≤1 cm	NR
**25**	36	Palpable mass. Cyclical pain	PF	1	4.3 CM	US, CT, MR	EX	DM	Free ≥1 cm	NR
**26**	38	Palpable mass. Cyclical pain	3 PF	1	2.3 CM	US, CT, MR	EX	DM	Free ≥1 cm	NR
**27**	34	Palpable mass. Cyclical pain	PF	1	3.1 CM	US, MR	EX	PP	Free ≤1 cm	NR
**28**	28	Palpable mass. Cyclical pain	PF	1	5.3 CM	US, MR	EX	PP	Free ≤1 cm	NR
**29**	43	Palpable mass. Cyclical pain	IVM	1	3.7 CM	US, CT, MR	EX	PP	Free ≤1 cm	NR
**30**	36	Palpable mass. Cyclical pain	PF	1	3 CM	US, CT, MR	EX	PP	Free ≤1 cm	NR
**31**	29	Palpable mass. Cyclical pain	PF	1	4.5 CM	US, MR	EX	PP	Free ≤1 cm	NR

## Data Availability

Data are contained within the article.

## Abbreviations

The following abbreviations are used in this manuscript:

AWE	Abdominal wall endometriosis
CS	Cesarean section
MRI	Magnetic resonance imaging
CT	Computed tomography

## References

[1] Liu G., Wang Y., Chen Y., Ren F. (2021). Malignant Transformation of Abdominal Wall Endometriosis: A Systematic Review of the Epidemiology, Diagnosis, Treatment, and Outcomes. Eur. J. Obstet. Gynecol. Reprod. Biol..

[2] Crump J., Suker A., White L. (2024). Endometriosis: A Review of Recent Evidence and Guidelines. Aust. J. Gen. Pract..

[3] Khan Z., Zanfagnin V., El-Nashar S.A., Famuyide A.O., Daftary G.S., Hopkins M.R. (2017). Risk Factors, Clinical Presentation, and Outcomes for Abdominal Wall Endometriosis. J. Minim. Invasive Gynecol..

[4] Falcone T. (2017). Clinical Management of Endometriosis. Semin. Reprod. Med..

[5] Koninckx P.R., Ussia A., Wattiez A., Zupi E., Gomel V. (2018). Risk Factors, Clinical Presentation, and Outcomes for Abdominal Wall Endometriosis. J. Minim. Invasive Gynecol..

[6] Allaire C., Bedaiwy M.A., Yong P.J. (2023). Diagnosis and Management of Endometriosis. Can. Med. Assoc. J..

[7] Ozel L., Sagiroglu J., Unal A., Unal E., Gunes P., Baskent E., Aka N., Titiz M.I., Tufekci E.C. (2012). Abdominal Wall Endometriosis in the Cesarean Section Surgical Scar: A Potential Diagnostic Pitfall. J. Obstet. Gynaecol. Res..

[8] Shim J.Y., Laufer M.R. (2020). Adolescent Endometriosis: An Update. J. Pediatr. Adolesc. Gynecol..

[9] Chapron C., Marcellin L., Borghese B., Santulli P. (2019). Rethinking Mechanisms, Diagnosis and Management of Endometriosis. Nat. Rev. Endocrinol..

[10] Falcone T., Flyckt-Rebecca R. (2018). Clinical Management of Endometriosis. Obstet. Gynecol..

[11] Hélage S., Rivière L., Buy J.N., Bordonné C., Préaux F., Just P.A., Aflak N., Rousset P., Dion É. (2024). MRI Classification of Uterosacral Ligament Involvement in Endometriosis: The Hôtel-Dieu Classification. Br. J. Radiol..

[12] Grigoriadis G., Roman H., Kalaitzopoulos D.R., Christoforidis N., Pados G., Daniilidis A. (2023). Diagnosis of Endometriosis by Transvaginal Ultrasound: An Online Survey of Gynecologists Practising in Greece. Cureus.

[13] de Venecia C., Ascher S.M. (2015). Pelvic Endometriosis: Spectrum of Magnetic Resonance Imaging Findings. Semin. Ultrasound CT MRI.

[14] Zanardi R., Del Frate C., Zuiani C., Bazzocchi M. (2003). Staging of Pelvic Endometriosis Based on MRI Findings versus Laparoscopic Classification According to the American Fertility Society. Abdom. Imaging.

[15] Leyendecker G., Wildt L., Laschke M.W., Mall G. (2023). Archimetrosis: The Evolution of a Disease and Its Extant Presentation: Pathogenesis and Pathophysiology of Archimetrosis (Uterine Adenomyosis and Endometriosis). Arch. Gynecol. Obstet..

[16] Habiba M., Guo S.W., Benagiano G. (2024). Are Adenomyosis and Endometriosis Phenotypes of the Same Disease Process?. Biomolecules.

[17] Kurman R.J., Shih I.M. (2010). The Origin and Pathogenesis of Epithelial Ovarian Cancer: A Proposed Unifying Theory. Am. J. Surg. Pathol..

[18] Tennfjord M.K., Gabrielsen R., Tellum T. (2021). Effect of Physical Activity and Exercise on Endometriosis-Associated Symptoms: A Systematic Review. BMC Womens Health.

[19] Practice Committee of the American Society for Reproductive Medicine (2014). Treatment of Pelvic Pain Associated with Endometriosis: A Committee Opinion. Fertil. Steril..

[20] Upadhyaya P., Karak A.K., Sinha A.K., Kumar B., Karki S., Agarwal C.S. (2010). Abdominal Wall Endometriosis. J. Nepal Med. Assoc..

[21] Rexhepi M., Asani L.V., Mulaki L., Koprivnjak K., Azemi M. (2023). Abdominal Wall Endometriosis at the Cesarean Section Scar. Prilozi.

[22] Costa J.E.F.R., Accetta I., Maia F.J.S., de Sá R.A.M. (2020). Abdominal Wall Endometriosis: Experience of the General Surgery Service of the Antônio Pedro University Hospital of the Universidade Federal Fluminense. Rev. Col. Bras. Cir..

[23] Vintilǎ D., Neacşu C.N., Popa P., Vlad N., Târcoveanu E., Georgescu S.O., Dǎnilǎ N. (2008). Abdominal Wall Endometriosis after Gynecologic Procedures: An under-Appreciated Diagnosis in General Surgery. Rev. Med. Chir. Soc. Med. Nat. Iasi.

[24] Yang E., Chen G.D., Liao Y.H. (2023). Spontaneous Abdominal Wall Endometriosis: A Case Report and Review of the Literature. Taiwan J. Obstet. Gynecol..

[25] Thanasa A., Thanasa E., Antoniou I.-R., Kontogeorgis G., Gerokostas E.-E., Kamaretsos E., Paraoulakis I., Simopoulou E., Mousia M., Thanasas I. (2024). Abdominal Wall Endometriosis: Early Diagnosis of a Rare Iatrogenic Complication Following Cesarean Section. Cureus.

[26] Rindos N.B., Mansuria S. (2017). Diagnosis and Management of Abdominal Wall Endometriosis: A Systematic Review and Clinical Recommendations. Obstet. Gynecol. Surv..

[27] Zhang P., Sun Y., Zhang C., Yang Y., Zhang L., Wang N., Xu H. (2019). Cesarean Scar Endometriosis: Presentation of 198 Cases and Literature Review. BMC Womens Health.

[28] Wasfie T., Gomez E., Seon S., Zado B. (2002). Abdominal Wall Endometrioma after Cesarean Section: A Preventable Complication. Int. Surg..

[29] Durairaj A., Sivamani H., Panneerselvam M. (2023). Surgical Scar Endometriosis: An Emerging Enigma. Cureus.

[30] Teng C.C., Yang H.M., Chen K.F., Yang C.J., Chen L.S., Kuo C.L. (2008). Abdominal Wall Endometriosis: An Overlooked but Possibly Preventable Complication. Taiwan. J. Obstet. Gynecol..

[31] Zhong Q., Qin S., Lai H., Yao S., Chen S. (2024). Risk Factors for Postoperative Recurrence of Cesarean Scar Endometriosis. AJOG Glob. Rep..

[32] Hocaoglu M., Turgut A., Ozdamar O., Aslan A., Demirer S., Usta A., Ekdeniz E., Karateke A. (2018). Abdominal Wall Endometriosis in Patients with a History of Cesarian Section. Ann. Ital. Chir..

[33] Horton J.D., DeZee K.J., Ahnfeldt E.P., Wagner M. (2008). Abdominal Wall Endometriosis: A Surgeon’s Perspective and Review of 445 Cases. Am. J. Surg..

[34] Ananias P., Luenam K., Melo J.P., Jose A.M., Yaqub S., Turkistani A., Shah A., Mohammed L. (2021). Cesarean Section: A Potential and Forgotten Risk for Abdominal Wall Endometriosis. Cureus.

[35] Doroftei B., Armeanu T., Maftei R., Ilie O.D., Dabuleanu A.M., Condac C. (2020). Abdominal Wall Endometriosis: Two Case Reports and Literature Review. Medicina.

[36] Foley C.E., Ayers P.G., Lee T.T. (2022). Abdominal Wall Endometriosis. Obstet. Gynecol. Clin. N. Am..

[37] Yarmish G., Sala E., Goldman D.A., Lakhman Y., Soslow R.A., Hricak H., Gardner G.J., Vargas H.A. (2017). Abdominal Wall Endometriosis: Differentiation from Other Masses Using Features. Abdom. Radiol..

[38] Welch B.T., Ehman E.C., VanBuren W.M., Cope A.G., Welch T.L., Woodrum D.A., Kurup A.N., Burnett T.L. (2020). Percutaneous Cryoablation of Abdominal Wall Endometriosis: The Mayo Clinic Approach. Abdom. Radiol..

[39] Bartlett D.J., Burkett B.J., Burnett T.L., Sheedy S.P., Fletcher J.G., VanBuren W.M. (2020). Comparison of Routine Pelvic US and MR Imaging in Patients with Pathologically Confirmed Endometriosis. Abdom. Radiol..

[40] Mahdavi A., Forouzannia S.A., Goudarzi E., Forouzannia S.M., Rafiei R., Yousefimoghaddam F., Rafiei N., Padmehr R. (2024). Radiofrequency Ablation: A Promising Treatment Method for Abdominal Wall Endometriosis. Cardiovasc. Interv. Radiol..

[41] Jouffrieau C., Cazzato R.L., Gabriele V., Faller E., Weiss J., Host A., Garnon J., Garbin O., Gangi A. (2023). Percutaneous Imaging-Guided Cryoablation of Endometriosis Scars of the Anterior Abdominal Wall. J. Minim. Invasive Gynecol..

[42] Cornelis F., Petitpierre F., Lasserre A.S., Tricaud E., Dallaudière B., Stoeckle E., Le Bras Y., Bouzgarrou M., Brun J.L., Grenier N. (2014). Percutaneous Cryoablation of Symptomatic Abdominal Scar Endometrioma: Initial Reports. J. Minim. Invasive Gynecol..

[43] Maillot J., Brun J.L., Dubuisson V., Bazot M., Grenier N., Cornelis F.H. (2017). Mid-Term Outcomes after Percutaneous Cryoablation of Symptomatic Abdominal Wall Endometriosis: Comparison with Surgery Alone in a Single Institution. Eur. Radiol..

[44] Cozzolino M., Magnolfi S., Corioni S., Moncini D., Mattei A. (2015). Abdominal Wall Endometriosis on the Right Port Site After Laparoscopy: Case Report and Literature Review. Ochsner. J..

[45] Denton G.W.L., Schofield J.B., Gallagher P. (1990). Uncommon Complications of Laparoscopic Sterilisation. Ann. R. Coll. Surg. Engl..

[46] Benedetto C., Cacozza D., de Sousa Costa D., Coloma Cruz A., Tessmann Zomer M., Cosma S., Trippia C.H., Santos Cavalcanti T.C., Alves Castro G.R., Kondo W. (2022). Abdominal Wall Endometriosis: Report of 83 Cases. Int. J. Gynecol. Obstet..

[47] Pavone M., Seeliger B., Alesi M.V., Goglia M., Marescaux J., Scambia G., Ianieri M.M. (2023). Initial experience of robotically assisted endometriosis surgery with a novel robotic system: First case series in a tertiary care center. Updat. Surg..

[48] Madar A., Crestani A., Eraud P., Spiers A., Constantin A., Chiche F., Furet E., Collinet P., Touboul C., Merlot B. (2025). Voiding dysfunction after surgery for colorectal deep infiltrating endometriosis: An updated systematic review and meta-analysis. Updat. Surg..

